# Effect of Baseline Dialysis and Plasma Exchange on Renal Prognosis in Patients With Antineutrophil Cytoplasmic Autoantibody–Associated Vasculitis

**DOI:** 10.1016/j.ekir.2025.09.005

**Published:** 2025-09-04

**Authors:** Maxime Vignac, Dorian Nezam, François Grolleau, Pauline Morel, Dimitri Titeca-Beauport, Stanislas Faguer, Alexandre Karras, Justine Solignac, Noémie Jourde-Chiche, François Maurier, Hamza Sakhi, Khalil El Karoui, Rafik Mesbah, Pierre Louis Carron, Vincent Audard, Didier Ducloux, Romain Paule, Jean-François Augusto, Julien Aniort, Aurélien Tiple, Cédric Rafat, Séverine Beaudreuil, Xavier Puéchal, Pierre Gobert, Ziad Massy, Catherine Hanrotel, Stéphane Bally, Nihal Martis, Cécile-Audrey Durel, Geoffroy Desbuissons, Pascal Godmer, Aurélie Hummel, François Perrin, Antoine Néel, Claire De Moreuil, Tiphaine Goulenok, Dominique Guerrot, Steven Grange, Aurélie Foucher, Alban Deroux, Alexandra Dervaux, Céline Guilbeau-Frugier, Anne Modesto-Segonds, Dominique Nochy, Laurent Daniel, Anissa Moktefi, Marion Rabant, Loïc Guillevin, Alexis Régent, Raphaël Porcher, Benjamin Terrier, French Vasculitis Study Group

**Affiliations:** 1 Service de Médecine Interne, Hôpital Cochin, Université Paris Cité, Paris, France; 2 Service de Néphrologie, Dialyse et Transplantation, CHU de Rouen, France; 3 Centre de Recherche épidémiologie et Statistiques, Université de Paris, Paris, France; 4 Service de dialyse et aphérèse, AURA Paris Plaisance, Paris, France; 5 Service de Néphrologie, CHU Amiens Picardie, Amiens, France; 6 Département de Néphrologie et Transplantation d’organes, Hôpital Rangueil, Toulouse, France; 7 Service de Néphrologie, Hôpital Européen Georges Pompidou, Université de Paris, Paris, France; 8 Centre de Néphrologie et Transplantation Rénale, Hôpital de la Conception (APHM), Marseille, France; 9 Hôpital Belle-Isle, Groupe Hospitalier Associatif UNEOS, Metz, France; 10 Service de Néphrologie et Transplantation, Centre de référence Maladie rare Syndrome Néphrotique Idiopathique, Hôpitaux Universitaires Henri Mondor, Créteil, France; 11 Institut National de la Santé et de la Recherche Médicale U955, Institut Mondor de Recherche Biomédicale, Universite Paris Est- Créteil, Créteil, France; 12 Unité de Néphrologie, Transplantation Rénale, Hôpital Tenon (Assistance Publique des Hôpitaux de Paris), Paris, France; 13 Service de Néphrologie, Centre Hospitalier, Boulogne-sur-mer, France; 14 Service de Néphrologie, Hôpital Nord, CHU Grenoble, Grenoble, France; 15 Service de Néphrologie, Dialyse et Transplantation, CHU Besançon, France; 16 Service de Médecine Interne, Hôpital Foch, Suresnes, France; 17 Service de Néphrologie, Dialyse, Transplantation, CHU, CHU Angers, Angers, France; 18 Service de Néphrologie, Dialyse et Transplantation rénale, CHU Gabriel Montpied, Clermont-Ferrand, France; 19 Service de Néphrologie, CHU Jacques Lacarin, Vichy, France; 20 Service de Néphrologie, Dialyse et Transplantation rénale, Hôpital Bicêtre, Le Kremlin Bicêtre, France; 21 Service de Médecine interne, Centre hospitalier Carpentras, Carpentras, France; 22 Département de Néphrologie, Hôpital Ambroise Paré, Boulogne Billancourt, France; 23 Service de Néphrologie, Dialyse et Transplantation rénale, Hôpital La Cavale Blanche, Brest, France; 24 Service de Néphrologie Dialyse, Centre Hospitalier Métropole Savoie, Chambery, France; 25 Service de Médecine Interne, CHU Nice, Nice, France; 26 Service de Médecine Interne, Hôpital Saint Joseph Saint Luc, Lyon, France; 27 Service de Néphrologie, Hôpitaux Privés de l’ouest Parisien, Trappes, France; 28 Service de Médecine Interne, CHBA site de Vannes, Vannes, France; 29 Service de Néphrologie et Transplantation Rénale, Hôpital Necker-Enfants Malades (Assistance Publique des Hôpitaux de Paris), Paris, France; 30 Service de Médecine Interne, CHU de Saint-Nazaire, France; 31 Service de Médecine Interne, CHU de Nantes, Nantes, France; 32 Service de Médecine Interne, CHRU de Brest, Brest, France; 33 Service de Médecine Interne, Hôpital Bichat (Assistance Publique des Hôpitaux de Paris), Paris, France; 34 Service de Néphrologie, CIC-CRB 1404, INSERM EnVi U1096, CHU Rouen, Rouen, France; 35 Service de Réanimation médicale, CHU Charles Nicolle, Rouen, France; 36 Service de Médecine Interne, CHU site Sud Saint-Pierre, Saint-Pierre, France; 37 Service de Médecine Interne, CHU de Grenoble, Grenoble, France; 38 Service d’anatomie et de cytologie pathologiques, Hôpital Nord, CHU d’Amiens, France; 39 Service d’anatomie pathologique et histologie-cytologie, Hôpital de Rangueil-Larrey, CHU Toulouse, Toulouse, France; 40 Service d’Anatomie et Cytologie Pathologiques, Hôpital Européen Georges Pompidou (Assistance Publique des Hôpitaux de Paris), Paris, France; 41 Service d’Anatomie et cytologie pathologiques, Hôpital La Timone (APHM), Marseille, France; 42 Assistance Publique des Hôpitaux de Paris, Department of Pathology, Groupe Hospitalier Henri-Mondor, Créteil, France; 43 Department of Pathology, Necker Hospital, Assistance Publique des Hôpitaux de Paris, Paris, France; 44 Université Paris Cité, Paris, France

**Keywords:** ANCA, hemodialysis, plasmapheresis, vasculitis

## Abstract

**Introduction:**

The identification of prognostic factors for renal failure in antineutrophil cytoplasmic autoantibody (ANCA)-associated vasculitis (AAV) remains a challenge. The benefit of plasma exchange (PLEX) has been questioned, and the target population remains to be defined. We investigated the outcome of patients requiring renal replacement therapy (RRT) at baseline and factors associated with their prognosis at 1 year.

**Methods:**

This retrospective multicenter study evaluated the 1-year composite end point of death or end-stage kidney disease (ESKD) in patients with biopsy-proven renal AAV involvement.

**Results:**

Of the 394 patients included, 105 (26.6%) were on dialysis at baseline. Of these, 60 (57.1%) reached the composite end point compared with 29 patients (10.0%) who were not on RRT at baseline (*P* < 0.001). On multivariate analysis, age and sex were not associated with the composite outcome (*P* = 0.945 and *P* = 0.154, respectively); however, myeloperoxidase (MPO)-ANCA was (odds ratio [OR]: 3.60; 95% confidence interval [CI]: 1.79–7.60), as was a high baseline histologic renal risk score (OR: 1.29; 95% CI 1.17–1.44). The most strongly associated factor remained the need for dialysis at baseline (OR: 10.91; 95% CI: 5.52–22.70). Of the 91 patients surviving after requiring dialysis at baseline, 45 were weaned from RRT (49.5%) at 1 year, and PLEX was independently associated with a reduced risk of the composite outcome (OR: 0.23, 95% CI: 0.05–0.80).

**Conclusion:**

MPO-ANCA, need for dialysis, and high histological renal risk score at baseline were associated with the 1-year composite end point of death or ESKD. Almost half of the patients on dialysis at baseline were off dialysis at 1 year, with a better prognosis in those who had received PLEX.

## Graphical abstract

**Figure:**
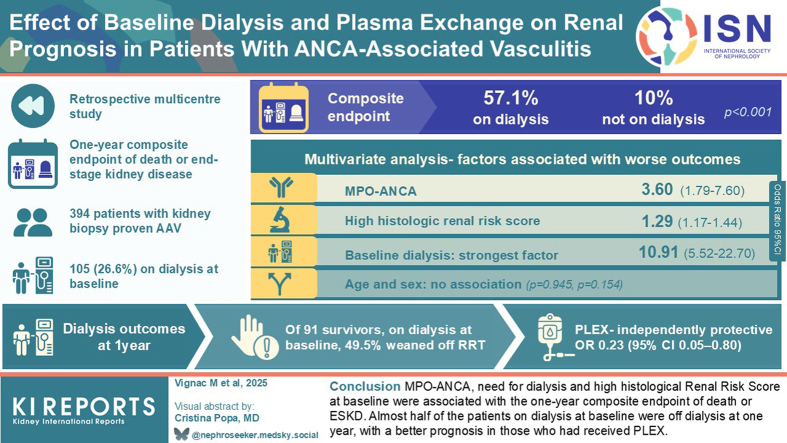

Renal involvement in AAV is more common in granulomatosis with polyangiitis and microscopic polyangiitis than in eosinophilic granulomatosis with polyangiitis. Nearly 80% of patients present with renal involvement during an AAV flare, and up to 20% of these patients develop ESKD.^1^ Therefore, renal involvement has a significant prognostic impact and has been included in the Five Factor Score.^2^ However, the renal characteristics of patients at baseline are heterogeneous and their prognostic trajectories are difficult to predict.^3^ It would be important to identify objective factors at baseline that are associated with prognosis and could guide treatment. Indeed, the consensus induction treatment for AAV with renal involvement remains high-dose glucocorticoids in combination with immunosuppressive treatment such as cyclophosphamide and/or rituximab.^4^, ^5^, ^6^ These regimens have been associated with better disease control but a high rate of infection, which remains a major cause of mortality in patients with AAV.^1^ In addition, the role of PLEX in these regimens remains unclear.

In the late 1990s, several studies with limited patient numbers suggested a benefit of PLEX in AAV with renal involvement.^7^, ^8^, ^9^ The MEPEX study, which enrolled 137 patients with serum creatinine > 500 μg/dl, showed a benefit with PLEX compared with methylprednisolone pulses at 3 months and 1 year, but no long-term benefit.^10^, ^11^, ^12^ The PEXIVAS study showed no benefit of PLEX efficacy in 704 patients with an estimated glomerular filtration rate (eGFR) < 50 ml/min per 1.73 m^2^ and/or diffuse pulmonary hemorrhage.^13^ Finally, the most recent meta-analysis on this topic suggest a benefit of PLEX on renal prognosis in the first year, but an increased rate of serious infection at 12 months and no effect on overall survival. Importantly, all these studies did not include the results of renal histologic characteristics in their analyses. Taken together, these results suggest that the target population that would benefit from PLEX remains to be defined.^14^

The objective of this study was to identify clinical, biological, and histological factors at baseline that are associated with the prognosis at 1 year of patients, according to their need for RRT. In addition, the association of PLEX use with prognosis in these subgroups was evaluated.

## Methods

### Patients and Study Design

We conducted a retrospective observational study involving patients screened from 31 French centers in nephrology or internal medicine departments.^15^ All patients had microscopic polyangiitis, granulomatosis with polyangiitis, or renal-limited vasculitis, according to the American College of Rheumatology criteria or the Chapel Hill Consensus Conference nomenclature.^16^^,^^17^ Patients diagnosed with AAV between May 1, 2004 and November 30, 2019 were included. Patients had to be positive for MPO-ANCA or proteinase 3 (PR3)-ANCA, and patients with nonspecific ANCA were not considered. All patients had rapidly progressive glomerulonephritis, defined as a rapid doubling of serum creatinine level and/or hematuria and/or glomerular proteinuria. Renal biopsy was performed at the time of diagnosis or within 1 month of the renal flare. Histopathological data were collected by the physician at each center and graded according to the Berden *et al.* classification and the 2018 ANCA renal risk score developed by Brix *et al.*^18^^,^^19^ No centralized histological evaluation was performed. eGFR was calculated using the Modification of Diet in Renal Disease method.^20^ Dialysis use was considered at baseline if initiated within 15 days. The Modification of Diet in Renal Disease–based eGFR for patients on dialysis was calculated at baseline to account for residual renal function and arbitrarily set to 0 during follow-up. No time limit was set for the use of PLEX. One-year outcomes were collected retrospectively, and patients with < 1 year of follow-up were excluded (Supplementary Figure S1).

### Outcomes

The primary composite outcome was all-cause death or ESKD defined by RRT (i.e., dialysis or transplantation) at 1 year. Secondary outcomes included the number of patients with an eGFR > 30 ml/min per 1.73 m^2^ at 12 months, the absolute value of the change in eGFR at 12 months, and the number of patients with a significant improvement in eGFR at 12 months defined as a delta eGFR > 15 ml/min per 1.73 m^2^.

### Statistical Analysis

Data are expressed as *n* (%), mean (SD) or median (interquartile range). Patient characteristics were compared according to the need for dialysis at baseline using appropriate statistical tests such as *t* test, Mann-Whitney-Wilcoxon test, chi-square test, or Fisher exact test. Primary and secondary end points were analyzed in the overall population and in the subgroup of patients on dialysis at baseline. For multivariate generalized linear models (i.e., linear and logistic), variables were selected based on the author's will for age or sex, reports from the literature, or the significance in univariate analysis. As an exploratory analysis, we analyzed patient characteristics and outcomes according to the use of PLEX or outcome at 1 year. Missing data were sparse and not handled. Statistical analyses were performed using RStudio 2023.06.0 and R 4.3.0 software (R Foundation for Statistical Computing).

The authors take the responsibility for the integrity of the data. The STROBE statement was used to report these observational data (Supplementary Table S2).^21^

### Ethics

The study received approval from the local Ethics Committee Institutional Review Board of the Cochin Hospital, Paris (Cochin-Port Royal Hospital, Paris, France; No. AAA-2020–08037) as was conducted in compliance with the Good Clinical Practice protocol and the Declaration of Helsinki principles.

## Results

### Patients’ Characteristics

Of the 394 patients included, 105 were on dialysis at baseline. In Table 1, we summarize patients’ characteristics according to the requirement of RRT at baseline. One hundred eighty-three patients were female (46.4%), with a higher proportion of patients not on dialysis at baseline (*P* = 0.045), and age did not differ between groups (62.8 [13.2] vs. 63.7 [14.6], *P* = 0.585). Vasculitis subtypes were similar between patients who were not on dialysis at baseline and those who were on dialysis at baseline, except a higher rate of PR3-ANCA in patients who were on dialysis at baseline (*P* = 0.025). Peak serum creatinine level was higher in patients requiring dialysis (700 [550–912] μmol/l) compared with patients not on dialysis (320 [200–420] μmol/l, *P* < 0.001). Patients on dialysis had a higher ANCA renal risk score (9.00 [7.00–10.00] vs. 5.00 [2.00–8.00], *P* < 0.001), and kidney damage was predominantly classified as sclerotic (35%) or crescentic (36%); whereas in patients not on dialysis, the predominant class was mixed (32%). Cyclophosphamide was used as induction therapy in 300 patients (76.1%) and rituximab in 94 patients (23.9%). PLEX was used in 170 patients (43.1%), mostly for patients on dialysis at baseline (*P* < 0.001).

**Table 1:** Patient characteristics according to dialysis use at baseline

Variables	Dialysis-free at baseline *n* = 289 (73.4%)	On dialysis at baseline *n* = 105 (26.6%)	*P*-value
Female, *n* (%)	143 (49.5%)	40 (38.1%)	0.045
Age, mean (SD), yr	62.8 (13.2)	63.7 (14.6)	0.585
Vasculitis, *n* (%)			0.240
GPA	102 (35.3%)	45 (42.9%)	
MPA	134 (46.4%)	47 (44.8%)	
Renal-limited vasculitis	53 (18.3%)	13 (12.4%)	
ANCA subtype, *n* (%)			0.025
PR3-ANCA	89 (30.8%)	45 (42.9%)	
MPO-ANCA	200 (69.2%)	60 (57.1%)	
Initial flare	277 (95.8%)	99 (94.3%)	0.512
Serum creatinine, median (EIQ), μmol/l	320 (200–420)	700 (550–912)	< 0.001
eGFR, median (EIQ), ml/min per 1.73 m^2^	16.0 (12.0–27.0)	5.00 (0.00–7.00)	< 0.001
Proteinuria, median (EIQ)^a^, g/24 h	1.80 (1.00–2.77)	2.50 (1.40–3.87)	0.002
ANCA Renal Risk Score, median [EIQ]	5.00 (2.00–8.00)	9.00 (7.00–10.0)	< 0.001
Berden classification, *n* (%) Overall			< 0.001
Sclerotic	59 (20.4%)	37 (35.2%)	0.002
Focal	63 (21.8%)	9 (8.57%)	0.003
Crescentic	74 (25.6%)	38 (36.2%)	0.039
Mixed	93 (32.2%)	20 (19.0%)	0.011
Induction immunosuppressive therapy, *n* (%)			0.076
CYC	202 (69.9%)	83 (79.0%)	
RTX	67 (23.2%)	12 (11.4%)	
Both	10 (3.46%)	5 (4.76%)	
Others	10 (3.46%)	5 (4.76%)	
Orotracheal intubation, *n* (%)	2 (0.71%)	12 (11.5%)	< 0.001
Plasmapheresis, *n* (%)	91 (31.5%)	79 (75.2%)	< 0.001
Number of PLEX, mean (SD)^b^	6.69 (1.59)	7.08 (2.68)	0.268
Number of PLEX, median (EIQ)^b^	7.00 (7.00–7.00)	7.00 (6.00–7.00)	0.954

ANCA, antineutrophil cytoplasmic autoantibody; CYC, cyclophosphamide; eGFR, estimated glomerular filtration rate; GPA, granulomatosis with polyangiitis; MPA: microscopic polyangiitis; MPO, myeloperoxidase; PLEX, plasma exchange; PR3, proteinase 3; RTX, rituximab.

a *N* = 272.

b *N* = 170.

### One-Year Prognosis

At 1 year, 60 of 105 patients on dialysis at baseline (57.1%) reached the primary composite end point, compared with 29 of 289 dialysis-free patients (10.0%) (Table 2). At 1 year, of the 105 patients on dialysis at baseline, 91 were still alive and 45 were weaned from RRT (49.5%). The percentage of patients with an eGFR > 30 ml/min per 1.73 m^2^ at 12 months was lower in patients on dialysis at baseline (30/105 [28.6%] vs. 169/289 [58.7%], *P* < 0.001). The median change in eGFR at 1 year did not significantly differ between patients who were dialysis-free at baseline and those who were on dialysis at baseline, nor did the proportion of patients who experienced a significant recovery (delta eGFR > 15 ml/min per 1.73 m^2^). Of the 305 patients alive and not requiring dialysis at 1 year (77.4%), 89 (29.2%) were classified as crescentic and 60 (19.7%) as sclerotic according to the Berden classification.

**Table 2:** One-year follow-up outcomes

Outcomes	Dialysis-free at baseline *n* = 289 (73.4%)	On dialysis at baseline *n* = 105 (26.6%)	*P*-value
Primary composite outcome, *n* (%)	29 (10.0%)	60 (57.1%)	< 0.001
Mortality at M12, *n* (%)	10 (3.46%)	14 (13.3%)	< 0.001
RRT at M12, *n* (%)	19 (6.57%)	46 (43.8%)	< 0.001
eGFR > 30 ml/min per 1.73 m^2^ at M12, *n* (%)	169 (58.7%)	30 (28.6%)	< 0.001
Delta eGFR > 15 ml/min per 1.73 m^2^ at M12, *n* (%)	133 (46.2%)	43 (41.0%)	0.356
Delta eGFR at M12, median (IQR)	13.0 (3.00–23.0)	0.00 (0.00–32.0)	0.303

eGFR, estimated glomerular filtration rate; IQR, interquartile range; M, month; RRT, renal replacement therapy.

### Factors Associated With the Primary Outcome

In multivariate analysis, neither age nor sex was associated with the primary composite outcome (OR: 1.00, 95% CI: 0.98–1.02 and OR: 1.55, 95% CI: 0.85–2.88, respectively) (Table 3). RRT at baseline was the strongest variable associated with 1-year outcome (OR: 10.91, 95% CI: 5.52–22.70). However, MPO-ANCA and elevated ANCA renal risk score were independently associated with worse prognosis (OR: 3.60, 95% CI: 1.79–7.60 and OR: 1.29, 95% CI: 1.17–1.44, respectively). Overall, PLEX was not significantly associated with the primary outcome in the overall cohort (OR: 0.86, 95% CI: 0.43–1.67).

**Table 3:** Logistic models predicting the primary composite outcome of death or ESKD at 1 year in the entire study population (*n* = 394)

Variables	Univariate		Multivariate	
	OR (95% CI)	*P*-value	OR (95% CI)	*P*-value
Age	1.00 (0.99–1.02)	0.672	1.00 (0.98–1.02)	0.945
Male	1.37 (0.85–2.22)	0.198	1.55 (0.85–2.88)	0.154
MPO-ANCA	2.06 (1.21–3.64)	0.010	3.60 (1.79–7.60)	< 0.001
ANCA Renal Risk Score	1.40 (1.28–1.54)	< 0.001	1.29 (1.17–1.44)	< 0.001
Dialysis	11.95 (7.01–20.87)	< 0.001	10.91 (5.52–22.70)	< 0.001
Plasma exchanges	2.10 (1.30–3.40)	0.002	0.86 (0.43–1.67)	0.659

CI, confidence interval; ESKD, end-stage kidney disease; MPO, myeloperoxidase; OR, odds ratio.

### Use of PLEX

**Figure 1:**
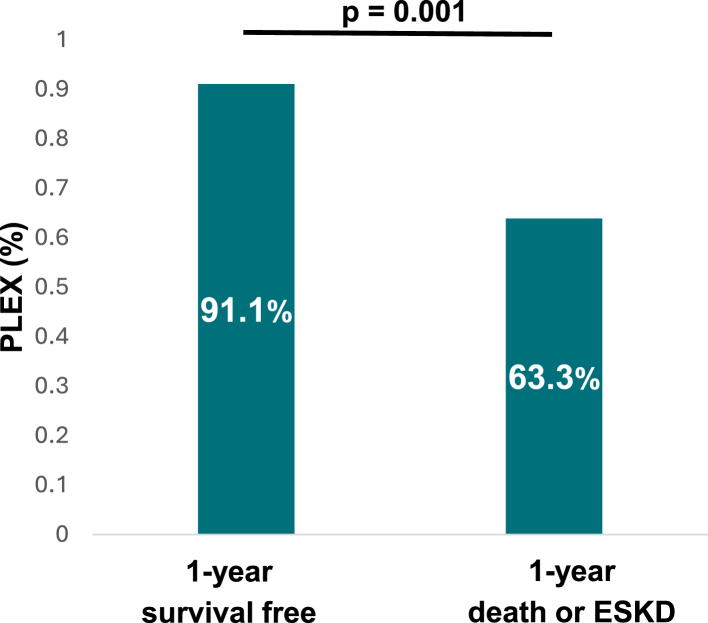
Prevalence of plasma exchange (PLEX) in patients on dialysis at baseline according to the 1-year primary composite outcome (*n* = 105).

Among patients on dialysis at baseline, 91.1% of those who were weaned from dialysis at 1 year had received PLEX, compared with only 63.3% of those who reached the composite outcome (*P* = 0.001) (Figure 1). Patients on dialysis at baseline receiving PLEX had a higher rate of PR3-ANCA (*P* = 0.019) and a lower ANCA renal risk score (*P* = 0.019) but no significant difference in the Berden classification (*P* = 0.099) (Supplementary Table S1). Similarly, patients not on dialysis at baseline receiving PLEX had greater renal involvement with higher serum creatinine level (*P* < 0.001), greater proportion of crescentic class in the Berden classification (*P* < 0.001), a trend for higher rate of PR3-ANCA (*P* = 0.056) and higher ANCA renal risk score (*P* = 0.119).

We then focused on the population of patients requiring dialysis at baseline. In this subpopulation, PLEX was independently associated with a better 1-year prognosis (OR: 0.23, 95% CI: 0.05–0.80, *P* = 0.029) (Table 4), whereas MPO-ANCA (OR: 3.10, 95% CI: 1.21–8.20, *P* = 0.019) and increased ANCA renal risk score (OR: 1.40, 95% CI: 1.16–1.73, *P* = 0.001) remained associated with a worse prognosis. In the linear model predicting the eGFR at 1 year, PLEX was associated with an improvement of 8.96 ml/min per 1.73 m^2^ (95% CI: 0.19–17.72, *P* = 0.045), whereas MPO-ANCA was associated with a decrease of 10.45 ml/min per 1.73 m^2^ (95% CI: −18.08 to −2.83, *P* = 0.008), and elevation of 1 point of ANCA renal risk score with a decrease of 3.49 ml/min per 1.73 m^2^ (95% CI: −4.89 to −2.10, *P* < 0.001) (Table 5).

**Table 4:** Multivariate logistic model representing the effect of plasma exchange on the primary composite outcome at 1 year in patients on dialysis at baseline (*n* = 105)

Variables	OR (95% CI)	*P*-value
Age	0.99 (0.96–1.03)	0.751
Male	0.71 (0.26–1.90)	0.497
MPO-ANCA	3.10 (1.21–8.20)	0.019
ANCA renal risk score	1.40 (1.16–1.73)	0.001
Plasma exchanges	0.23 (0.05–0.80)	0.029

ANCA, antineutrophil cytoplasmic autoantibody; CI, confidence interval; OR, odds ratio; MPO, myeloperoxidase.

**Table 5:** Multivariate linear model representing the effect of plasma exchange on the change in absolute value of eGFR at 1 year in patients on dialysis at baseline (*n* = 105)

Variables	Estimates (95% CI)	*P*-value
Age	−0.13 (−0.39 to 0.13)	0.311
Male	−4.10 (−11.70 to 3.50)	0.287
MPO-ANCA	−10.45 (−18.08 to −2.83)	0.008
ANCA renal risk score	−3.49 (−4.89 to −2.10)	< 0.001
Plasma exchanges	8.96 (0.19–17.72)	0.045

ANCA, antineutrophil cytoplasmic autoantibody; CI, confidence interval; OR, odds ratio; MPO, myeloperoxidase.

## Discussion

In this large, retrospective, observational cohort study of patients with AAV renal involvement, we found that nearly half of patients on dialysis at baseline were off dialysis at 1 year, which is slightly higher than previously reported.^22^, ^23^, ^24^ In contrast, only 10% of patients who were dialysis-free at baseline reached the composite outcome of death or RRT at 1 year. We identified the need for dialysis at baseline as the most important prognostic factor, in addition to MPO-ANCA positivity and histological ANCA renal risk score at baseline, which were also independently associated with prognosis. In contrast, age was not associated with renal prognosis either at baseline (i.e., dialysis use) or at 1 year (i.e., death or RRT). In addition, PLEX was used in patients with higher renal involvement (i.e., serum creatinine level and histological classifications) in patients not on dialysis at baseline whereas no such difference was found in patients on dialysis at baseline. We observed a higher rate of dialysis use among patients with PR3-ANCA positivity in both groups. Finally, PLEX was independently associated with a better prognosis specifically in patients on dialysis at baseline, but no significant association was found in the overall cohort.

The potential benefit of intensive treatment for patients with severe involvement and sclerotic kidney damage at biopsy is often debated because of the lack of expected recovery. In our cohort, a significant proportion of patients with sclerotic histologic classification at baseline recovered renal function at 1 year. These results strongly support maximal treatment even in patients with sclerotic damage (i.e., > 50% global sclerotic glomeruli) despite physician reluctance.

The benefit of PLEX in AAV remains uncertain because the last meta-analysis on this topic produced conflicting results.^14^^,^^25^^,^^26^ This may be because of heterogeneity in selection criteria, such as different thresholds for renal failure. Overall, it appears that some patients may benefit from PLEX; however, the effects are diluted in larger study populations. In this context, we present here the association of PLEX with a better prognosis specifically in patients with AAV requiring dialysis at baseline, independent of known confounders, in particular kidney histopathology. The need for dialysis at baseline may serve as a pragmatic selection criterion for patients who may benefit from PLEX. Compared with serum creatinine, this is a strong criterion with limited subjectivity or time variability.

However, our results differ from other studies, including the PEXIVAS study. This study performed a subgroup analysis of patients who were on dialysis at baseline or had a serum creatinine level > 5.6 mg/dl.^13^ The authors showed a nonstatistically significant trend toward a benefit of PLEX on the composite outcome of death or ESKD in this subgroup. More recently, in a retrospective cohort study with a limited number of patients with AAV requiring dialysis at baseline, PLEX was not significantly associated with a better prognosis at 1 year.^27^ However, our study was conducted in a real-world setting with histology as a confounding factor, which may explain some of the discrepancies.

Our study has several strengths. We report the outcome of a large number of patients. In addition, the important cornerstone of our study is that the analysis and models included histological data, which is known to be highly associated with prognosis. However, our study is limited in that we can only present associations between variables without inferring direct causality. The results are based on a retrospective design with limited follow-up, and we anticipate subsequent selection bias, particularly in patients on dialysis and in the use of PLEX. In addition, expected adverse events such as infection rates were not reported. However, we believe that these biases would affect our results quantitatively rather than qualitatively. Finally, patients were enrolled from several centers in France between 2004 and 2019, before the approval of new drugs such as C5aR inhibitors.

In conclusion, despite the high burden of AAV and dialysis, > 40% of patients requiring dialysis at baseline are alive and dialysis-free at 1 year. The need for dialysis at baseline was associated with poor survival and renal prognosis, such as MPO-ANCA and renal histological data. Among patients on dialysis at baseline, the use of PLEX was associated with a better prognosis, independent of the identified confounders. Therefore, the need for dialysis at baseline in AAV appears to be a pragmatic selection criterion for patients who may benefit from PLEX and merits investigation.

## Disclosure

JA, J-FA, PLC, CDM, LG, RM, and XP participated in the PEXIVAS trial. VA reports receiving honoraria from Addmedica, Alnylam, and Travere; and scientific advisor or membership with Addmedica, Alnylam, and Travere. J-FA reports honoraria from Sanofi and Therakos/Mallinckrot. SB reports honoraria from Alexion Pharma France for an intervention as a speaker (about TMA and atypical HUS). C-AD reports receiving fees from Vifor Pharma and honoraria from the scientific board with Vifor Pharma France. KEK reports research funding from Amgen, Otsuka, and Sanofi; and honoraria from Alexion and Otsuka. SF reports consultancy agreements with Abionyx Pharma and other interests or relationships with Vifor Pharma (symposium speaker). PG reports other interests or relationships with Société Nationale Française de Médecine Interne. SG reports scientific advisor or membership with Alexion. LG reports consultancy agreements with Behringer, Biogen, Certara, GSK, Lilly, Novartis, Novo Nordisk, Roche, Sanofi, Seattle Genetics, and UCB; research funding from Roche, which provided free rituximab for an academic study sponsored by the French Ministry of Health; honoraria for consultancies for Behringer, Biogen, Certara, GSK, Lilly, Novartis, Novo Nordisk, Roche, Sanofi, Seattle Genetics, and UCB; and other interests or relationships as a member of the board of the Haute Autorité de Santé and president of the Transparency Commission on drugs (duty finished in 2016). NJ-C reports research funding from Fresenius Medical Care for the CINEVAS study (NCT03635385) and honoraria from Vifor. AK reports consultancy agreements with Alnylam, GSK, Novartis, and Vifor; honoraria from AbbVie, Amgen, AstraZeneca, Gilead, GSK, Pfizer, and Roche Pharmaceuticals; and scientific advisor or membership with Novartis. NM reports ownership interest with Merck; honoraria from Sanofi Genzyme; and speakers bureau for Sanofi Genzyme. ZM reports research funding from Amgen, Baxter, Fresenius Medical Care, Genzyme-Sanofi, GlaxoSmithKline, Lilly, Merck Sharp and Dohme-Chibret, and Otsuka; government support for the CKD REIN project and experimental projects; honoraria to charities or for travel from Baxter and Genzyme-Sanofi; and scientific advisor or membership with Journal of Nephrology, Journal of Renal Nutrition, Kidney International, Nephrology Dialysis Transplantation, and Toxins. XP reports research funding from ChemoCentryx as an investigator in studies evaluating CCX168 in ANCA-associated vasculitis, InflaRx as an investigator in studies evaluating IFX1 in ANCA-associated vasculitis, and Roche Pharma as an investigator in academic studies of ANCA-associated vasculitis for which rituximab was provided by Roche Pharma. MR reports being scientific advisor or membership with Travere. CR received travel grants from Fresenius and honoraria from M3, Qualworld, and Sermo. BT received some consulting fees and/or grants from AstraZeneca, Bristol-Myers Squibb, GlaxoSmithKline, Grifols, LFB, Lilly, Roche/Chugaï, Terumo BCT, and Vifor Pharma outside of the submitted work. All the other authors declared no competing interests.

## Supplementary Material

Supplementary File (PDF) **Figure S1.** Flowchart of the study. **Table S1.** Patient characteristics by use of plasma exchange. **Table S2.** STROBE checklist.
